# Use of Nitrate of Silver for White Swellings, Hydrarthrosis, and Venereal Bubo

**Published:** 1849-10

**Authors:** 


### SELECTIONS

FROM

### AMERICAN AND FOREIGN JOURNALS.

---

*Use of Nitrate of Silver for White Swellings, Hydrarthrosis, and Venereal Bubo.*—M. Decaisne, military surgeon at Anvers, has published, in the Archives de Médecine Militaire, some observations on this subject; two remarkable cases are given. One of the patients was a man of 27 years of age, and of a lymphatic temperament, who, on the night of the 5th of February, 1847, felt a severe pain in the right knee; swelling soon followed, and the patient was unable to use the limb. Every means were had recourse to for his relief—antiphlogistics, baths, calomel and opium, blisters, iodine, mercurial ointment, compression and douches, without any effect in arresting its progress into regular white swelling. In the month of July, M. Decaisne began the use of an ointment of nitrate of silver, when the knee was double its natural size, was so tender that the patient dreaded its being touched, all motion in the joint impossible, and three fistulous openings at the inner side of the joint; amputation appeared inevitable; and in this very unfavorable aspect of affairs, it was ordered to employ friction twice a day of an ointment composed of one gros (59.1 grains) of the salt of silver to an ounce of lard; about two gros of the ointment were used at each application. Under this treatment the pain sensibly abated in a few days, the swelling gradually diminished, and in about a month the improvement, in every respect, was considerable. During the month of August, the proportion of the nitrate of silver to the lard was increased to a gros and a half or two gros to the ounce of lard, and at length, at the end of the month, the cure was complete, and the young man only experienced a slight stiffness in bending the knee.

The second case was that of a young boy, attacked with a white swelling of the radio-carpal articulation. Previously to the employment of the ointment of the nitrate of silver in this case, a number of the more active remedies had been tried in vain; the swelling was considerable, and it was necessary to open a large abscess near the articulation. After using the ointment of the nitrate of silver for two months the amelioration was considerable, or rather the cure was completed.

After giving the above cases, the Journal de Médecine adds the following remarks: Other cases, where the salt of silver in the form of ointment have been recently published in the Archives de la Médecine Belge, by Professor Uytterhoeven; he used it in a great number of cases, but all those he details were dropsy of the joints, not white swelling. The ointment of nitrate of silver possesses a resolutive action upon those serous swellings of joints. This therapeutic agent should not be employed until the inflammatory stage has passed.

In making the ointment it is necessary to dissolve the nitrate of silver in water before incorporating it with the lard, to prevent the rubefacient or cauterizing effect of the metallic salt on the skin, or the formation of vesicles, which without this precaution would be inevitable. Generally smart pain, but transient, is experienced on the application of this remedy at the place on which it is rubbed.

The power of this ointment to resolve venereal buboes has been experienced in the practice of M. Lutens; he dissolved a drachm of the salt in a sufficient quantity of distilled water, and then mixed it with an ounce of lard. His mode of using it is this,—about two drachms of the ointment are used at each rubbing; after three or four days the skin becomes black and shining; instead of suspending the treatment until the epidermis desquamates fully, the scales are detached with a nail, or a spatula, and the frictions immediately recommenced. These frictions never occasion pain, but sometimes a slight uneasiness. M. Lutens uses this ointment also in glandular swellings of the neck and groin, and in all stages of bubo. —*Dublin Med. Press.*

